# Portuguese Family Physicians’ Awareness of Diagnostic and Laboratory Test Costs: A Cross-Sectional Study

**DOI:** 10.1371/journal.pone.0137025

**Published:** 2015-09-10

**Authors:** Luísa Sá, Cristina Costa-Santos, Andreia Teixeira, Luciana Couto, Altamiro Costa-Pereira, Alberto Hespanhol, Paulo Santos, Carlos Martins

**Affiliations:** 1 Family Medicine Unit, Social Sciences and Health Department of the Faculty of Medicine of Porto, Porto, Portugal; 2 Family Health Unit Nova Via, Porto, Portugal; 3 Centre for Research in Health Technologies and Information Systems (CINTESIS) and Information Sciences and Decision on Health Department (CIDES), Faculty of Medicine, University of Porto, Porto, Portugal Al. Prof. Hernâni Monteiro 4200–319 Porto, Portugal; Central South University, CHINA

## Abstract

**Background:**

Physicians’ ability to make cost-effective decisions has been shown to be affected by their knowledge of health care costs. This study assessed whether Portuguese family physicians are aware of the costs of the most frequently prescribed diagnostic and laboratory tests.

**Methods:**

A cross-sectional study was conducted in a representative sample of Portuguese family physicians, using computer-assisted telephone interviews for data collection. A Likert scale was used to assess physician’s level of agreement with four statements about health care costs. Family physicians were also asked to estimate the costs of diagnostic and laboratory tests. Each physician’s cost estimate was compared with the true cost and the absolute error was calculated.

**Results:**

One-quarter (24%; 95% confidence interval: 23%–25%) of all cost estimates were accurate to within 25% of the true cost, with 55% (95% IC: 53–56) overestimating and 21% (95% IC: 20–22) underestimating the true actual cost. The majority (76%) of family physicians thought they did not have or were uncertain as to whether they had adequate knowledge of diagnostic and laboratory test costs, and only 7% reported receiving adequate education. The majority of the family physicians (82%) said that they had adequate access to information about the diagnostic and laboratory test costs. Thirty-three percent thought that costs did not influence their decision to order tests, while 27% were uncertain.

**Conclusions:**

Portuguese family physicians have limited awareness of diagnostic and laboratory test costs, and our results demonstrate a need for improved education in this area. Further research should focus on identifying whether interventions in cost knowledge actually change ordering behavior, in identifying optimal methods to disseminate cost information, and on improving the cost-effectiveness of care.

## Introduction

The Portuguese National Health Service (NHS) is a large health care organization that serves almost 10 million beneficiaries and contains a strong core of primary care physicians; 87.1% of Portuguese have family physicians[1]. According to data from the Organisation for Economic Co-Operation and Development (OECD), in 2012 Portugal spent 9.5% of its gross domestic product on healthcare costs, which is slightly above the OECD average of 9.3%[2]. However, Portugal spends much less than other European countries (e.g., the Netherlands [11.8%], France [11.6%], and Switzerland [11.4%]) and the United States (16.9%)[2]. In terms of health policy and in the context of significant budget constraints related to the current economic and financial situation, Portugal requires increased accuracy and accountability in the area of health management.

According to the 2009/2010 activity report of the Central Administration of the Health Service, laboratory examinations and diagnoses in ambulatory care constitute a significant proportion on total health expenses and represent about 8% of the expenses of the Portuguese NHS[3]. Units of primary health care are the most representative entities in terms of costs of prescriptions[3]. Analytical tests are the most prescribed laboratory examinations and diagnoses, followed by radiological tests[3].

Several studies have found frequent and excessive test ordering in most clinical environments, including primary care. [4][5] Many patients request or receive “routine” but unnecessary medical tests. The majority of Portuguese adults believe that they should utilize a great number of healthcare services on a nearly annual basis, and most actually follow this schedule. This finding indicates a tendency toward the overuse of resources[6]. Furthermore, these tests generally have little impact on patient care and in some cases can be harmful [5].

For these reasons, family physicians are under pressure to decrease the cost of diagnostic and laboratory test prescriptions and to perform a more cost-effective clinical practice[7]. These parameters are continuously assessed through performance indicators for each primary health care unit and for each family physician by the Central Administration of the Health Service [8][9]. The Portuguese Health Ministry recently published a set of clinical guidelines in order to promote more rational prescriptions of laboratory and diagnostic tests, and to improve the quality of care and its cost-effectiveness [7]. A second strategy that was recently implemented by the Ministry of Health was to incorporate a button with the euro currency (€) symbol within the laboratory test ordering system; clicking on the button allows the prescribing physician to know the exact cost of each test. However, we suspect that consultation regarding this information is low.

Physicians’ ability to make cost-effective decisions is likely limited by their poor understanding of health care costs[10][11][12][13][14]. A systematic review of physician knowledge of diagnostic and nondrug therapeutic costs that was performed in 2008 and included 14 studies found that only 33% of physician estimates were within 20% to 25% of the true costs, and that only 50% were within 50% of the true cost [12]. Previous studies [10,11] indicate that physicians do not receive adequate education about cost, have limited access to cost information, would like to receive more information, and feel that improved knowledge would change their ordering behavior.

There is evidence that physicians reduce costs when they have information such information[13][14][15][16][17][18], and some researchers have begun to investigate different strategies for promoting costs awareness [19][20][21][22][23].

There are no studies in Portugal about physicians’ knowledge about healthcare costs, and before implementing additional strategies, it is important to assess their current knowledge and to determine whether they are likely to benefit from additional education in this field.

The aim of this study is to assess whether Portuguese family physicians are aware of the costs of the most frequently prescribed diagnostic and laboratory tests.

## Methods

### Study design

A cross-sectional study was conducted among a representative sample of Portuguese family physicians using computer-assisted telephone interviews for data collection.

### Setting

The defined target population was the population of Portuguese family physicians working in the NHS. Data collection occurred between April 1, 2012 and October 12, 2012.

### Selection criteria

All medical doctors working as family physicians in a NHS Primary Care Health unit were eligible. There were no exclusion criteria.

### Survey sampling methods and sample size

Given that no official database of family physicians working in the NHS was available, a stratified cluster sampling design was used to obtain a representative sample of Portuguese family physicians. The Portuguese NHS is administratively divided into five regional health administrations. We considered each of these as a stratum and randomly selected a proportion of the primary health care units from each regional health administration. Each primary health care unit was a cluster, and we randomly selected one-third of the family physicians working in each unit.

According to the Portuguese National Institute of Statistics, in 2010 there were 5,273 family physicians working in the NHS [24]. According to the list of primary health care units provided by the Ministry of Health, there were 902 units (i.e., with an average of 5.85 family physicians per unit).

To determine the sample size, we used the recommended protocol for this type of sampling design [24], which has the following steps:

1. Set the desired precision of the estimates.
2. Set the number of family physicians (i.e., secondary sampling units) to be selected in each primary health care unit (i.e., primary sampling units or "clusters").
3. Set the number of primary health care units to be selected for the sample (i.e., primary sampling units or "clusters").

With regard to the required precision for the estimation of proportions, the margin of error (half-width of the 95% confidence interval) was set at no greater than 7.5%.

The definition of the number of family physicians to be selected in each primary health care unit took into account the minimization of the variance of the desired estimates for a given fixed cost of the sampling procedures. We selected an average of two family physicians in each primary health care unit, which corresponded to nearly one-third of the family physicians in each primary health care unit.

Finally, to define the number of primary health care units to be selected for the sample, we followed an approximation method based on an estimate of the design effect. [25]

According to our calculations, the size of our sample should range from 174 to 301 family physicians. Due to financial constraints, the final sample size defined for this study consisted of 180 family physicians distributed in 90 primary care health units, proportionately and randomly selected from each regional health administration, as shown in Table 1.

**Table 1:** Primary Health Care Units by Regional Health Administrations in the Portuguese National Health Service.

	Number of Primary Health Care Units in the Portuguese National Health Service	Number of Primary Health Care Units (clusters) selected for our sample
Northern Regional Health Administration	381	38
Centre Regional Health Administration	65	6
Lisbon and Tagus Valley Regional Health Administration	380	38
Alentejo Regional Health Administration	57	6
Algarve Regional Health Administration	19	2
**Total**	**902**	**90**

### Quality control

The interviewers had extensive experience in computer-assisted telephone interviewing and were adequately trained in and prepared for the application of the study questionnaire. A pilot test was conducted in order to assess the time required to complete the questionnaire and to assess questionnaire language and comprehension issues. A data collection supervisor supervised all interviews. At least one study coordinator randomly supervised 20% of the interviews.

### Instruments and methods for data collection

In order to achieve the best response rate and to avoid selection bias, we wrote to all family physicians working at the selected primary health care units by electronic mail and included information about the study, ethical and administrative authorizations, and possible future telephone contact. Simultaneously, we sent electronic mail correspondence to Portuguese family physician mailing groups informing them about this study.

The first telephone contact was made to the family physicians selected at each primary health care unit to ask for consent and identify preferred dates and times to conduct the interview. Since the contact was made by telephone, only oral consent was obtained. Data collection was carried out between March 29, 2012 and October 12, 2012, using computer-assisted telephone interviews. A structured questionnaire containing the following three sections was used: (1) introductory section presenting study aims and consent form, (2) the research instrument, and (3) socio-demographic data including age, gender, practice location (i.e., urban or rural as defined by the participant), and professional qualifications.

In the first part of the research section, we used a Likert scale (1–5) to assess family physicians’ level of agreement with four statements about health care costs. The four statements were:

- The cost of a diagnostic and laboratory test influences my decision when ordering it.
- I have adequate knowledge of diagnostic and laboratory test costs.
- I received adequate education on the diagnostic and laboratory test costs.
- I have adequate access to information on the diagnostic and laboratory test costs.

In the second part of the interview, we asked family physicians to spontaneously estimate the total costs of laboratory examinations and diagnostic tests in euros without consulting any other source of information. The total cost of a test includes the portion that is paid by the NHS and a charge that is paid by the patient. This was explained to the responders. The actual prices for laboratory examinations and diagnoses were obtained from the Central Administration of the Health Services, with the prices used as of January 1, 2012 [26]. In Portugal, patients obtain the tests’ prescription in the NHS medical consultation and then may choose a contracted laboratory to perform the tests. These contracted laboratories are private entities that have an existing contract with the Ministry of Health, allowing them to perform and to be paid for the tests prescribed by NHS physicians.

We inquired about the following diagnostic and laboratory tests: hemogram, platelets, glucose, total cholesterol, high-density lipoprotein (HDL) cholesterol, triglycerides, creatinine, urea, aspartate aminotransferase (AST), alanine aminotransferase (ALT), gamma-glutamyl transferase (GGT), proteinogram, sedimentation rate, ionogram, urine II, fecal occult blood test (FOBT; 3 samples), cervical cytology (i.e., Pap smear), mammography, bone densitometry, abdominal computed tomography, chest x-ray, breast ultrasound, abdominal ultrasound, pelvic ultrasound, prostate ultrasound, upper digestive endoscopy, total colonoscopy, flexible sigmoidoscopy, carcinoembryonic antigen (CEA), prostate-specific antigen (PSA), electrocardiogram (ECG), ECG stress test, and echocardiogram.

This research section was an authorized adaptation of the questionnaire used in a Canadian study[10).

### Statistical analysis

Statistical analysis was performed using the Statistical Package for the Social Sciences Version 21.0 for Windows (SPSS, Chicago, IL).

Each physician’s estimate of the cost was compared with the true cost, and the absolute error was calculated. In order to achieve comparable results with previous studies, we calculated the proportion of estimates accuracy within 25%[10], [11] and 50%[10][12] of the true cost. For each item, the median, minimum and maximum estimated cost, and the proportion of family physicians that over- or underestimated the true cost were determined.

To study if there was any association among cost estimates of each medical test and gender, chi-square tests were performed (one for each medical test). The same methodology was applied to the age category and workplace (rural or urban).

Only significant results were presented in the results section. This part of the study may be considered exploratory, since data were collected with a different objective and not with a pre-specified key hypothesis on gender, category age or workplace. Therefore, multiple test adjustment was not strictly required[27].

To determine if the number of tests in which a doctor correctly estimated costs to within 25% was different according to the level of their agreement with different statements about health care costs, a Kuskal-Wallis test was used.

In all analyses, a p < .05 level was considered significant.

### Ethical considerations

This study was approved by the Northern Regional Health Administration Ethics Committee for Health. All participants provided their verbal informed consent at the beginning of the telephone interview. The need for a written assigned document of consent was waived by the ethics committee because interviews were conducted by telephone (i.e., without the physical presence of participants). Participants were informed about the estimated duration of the interview, confidentiality was assured, and the voluntary nature of participation was emphasized. Participants were informed that they could interrupt their participation at any time. The interviews were not recorded and participants did not receive any form of compensation. To standardize the process of obtaining informed consent, the interviewers read the text of the informed consent.

## Results

In the 90 selected primary healthcare units, 255 randomly selected family physicians were invited to participate and 244 agreed to answer our questionnaire, resulting in a response rate of 96%. The mean duration of the interview was 15 minutes. Most of the participants were between 30 and 59 years old, and 61% were women. In Table 2, we present the demographic description of our study sample characteristics.

**Table 2:** Study sample demographic characteristics.

Sample characteristics	n	(%)
**Age** (years)		
20 to 29	1	(1)
30 to 39	33	(13)
40 to 49	44	(18)
50 to 59	155	(64)
60 to 69	11	(4)
**Gender**		
Male	95	(39)
Female	149	(61)
**Regional Health Administration**		
North	82	(34)
Center	17	(7)
Lisbon and Tagus Valley	130	(53)
Alentejo	11	(4)
Algarve	4	(2)
**Workplace**		
Urban	157	(64)
Rural	87	(36)
**Professional situation**		
Specialized in Family Medicine/General Practice	241	(99)
Not specialized, but working as Family Doctor	3	(1)
**Duration of work in Family Medicine/General Practice (years)**		
Fewer than 2 years	5	(2)
2 to 10 years	35	(15)
More than 10 years	201	(83)

**Fig 1:**
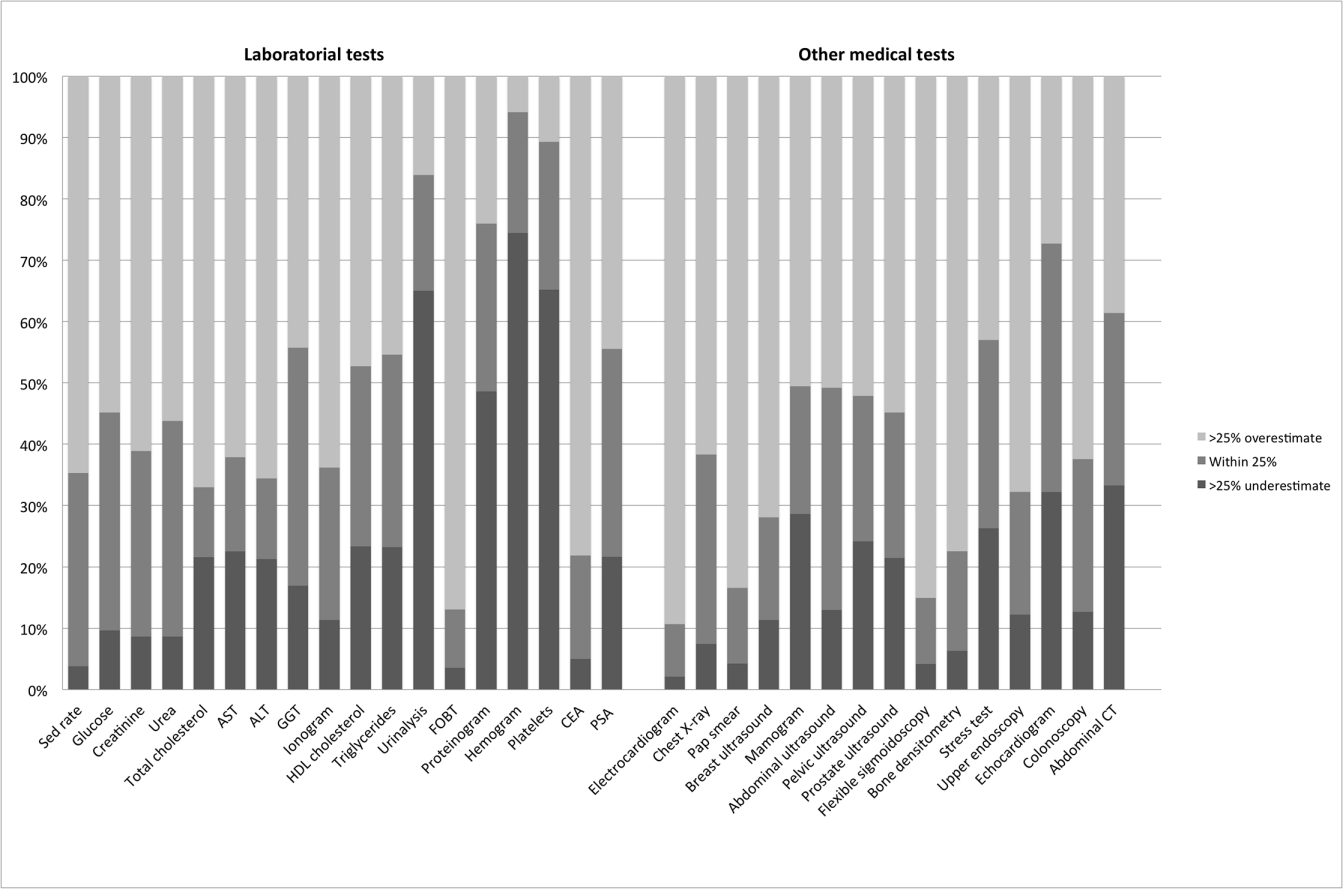
Percentage of family physicians that underestimated, correctly estimated, and overestimated the true cost associated each test to within 25% of true cost.

The distribution of family physicians that underestimated, correctly estimated, and overestimated the cost associated within each test to within 25% of true cost, as broken out by laboratory tests and other medical tests, and shown in ascending order of cost, is provided in Fig 1. Table 3 summarizes the items surveyed. The “don’t know” or “don’t want to answer” rate was 34% (Table 3).

**Table 3:** Real and attributed cost of diagnostic and laboratory tests.

	Don’t know or don’t want to answer (n, (%))	Real Cost (€)	Correct estimates* (%)	Over estimates* (%)	Under estimates* (%)	Median cost estimate (€)	Minimum estimate (€)	Maximum estimate (€)
***LABORATORY TESTS***								
**Sed rate**	60 (33)	0,95	32	65	4	2,00	0,40	50,00
**Glucose**	58 (31)	1,20	36	54	10	2,00	0,20	50,00
**Creatinine**	59 (32)	1,30	30	61	9	2,00	0,08	15,00
**Urea**	59 (32)	1,30	35	56	9	2,00	0,50	12,00
**Total Cholesterol**	59 (32)	1,40	11	67	22	2,00	0,50	32,50
**AST**	62 (34)	1,40	15	62	23	2,00	0,50	12,00
**ALT**	61 (33)	1,40	13	66	21	2,00	0,08	15,00
**GGT**	61 (33)	1,60	39	44	17	2,00	0,50	15,00
**Ionogram**	59 (32)	1,60	25	64	11	3,00	0,50	20,00
**HDL Cholesterol**	60 (33)	1,90	29	47	23	2,00	0,70	15,00
**Triglycerides**	59 (32)	1,90	31	45	23	2,00	0,50	20,00
**Urinalysis**	58 (31)	2,90	19	16	65	2,00	0,50	14,00
**FOBT**	76 (45)	3,00	10	79	4	10,00	2,00	50,00
**Proteinogram**	61 (33)	4,80	27	24	49	4,00	0,70	100,00
**Hemogram**	56 (30)	5,00	20	6	75	2,00	0,30	20,00
**Platelets**	57 (31)	5,00	24	11	65	3,00	0,50	30,00
**CEA**	84 (53)	8,90	17	78	5	20,00	3,00	150,00
**PSA**	64 (36)	9,50	34	44	22	10,00	1,50	400,00
***OTHER MEDICAL TESTS***								
**Electrocardiogram**	56 (30)	3,87	9	89	2	10,00	1,50	50,00
**Chest X-ray**	56 (30)	4,62	31	62	7	10,00	1,50	100,00
**Pap smear**	57 (31)	5,42	12	83	4	15,00	2,00	150,00
**Breast ultrasound**	59 (32)	14,30	17	72	11	25,00	5,00	100,00
**Mammogram**	66 (37)	20,09	32	51	29	30,00	2,00	200,00
**Abdominal ultrasound**	59 (32)	20,12	36	51	13	30,00	5,00	100,00
**Pelvic ultrasound**	58 (31)	20,16	24	52	24	30,00	5,00	100,00
**Prostate ultrasound**	58 (31)	20,16	24	55	22	30,00	5,00	100,00
**Flexible sigmoidoscopy**	77 (46)	22,64	11	85	4	50,00	10,00	400,00
**Bone densitometry**	71 (41)	23,22	16	78	6	40,00	2,00	250,00
**Stress test**	58 (31)	27,55	31	43	26	30,00	7,00	200,00
**Upper endoscopy**	64 (36)	34,31	20	68	12	52,50	10,00	200,00
**Echocardiogram**	61 (33)	40,70	40	27	32	44,50	10,00	200,00
**Colonoscopy**	63 (35)	51,21	25	62	13	80,00	17,00	300,00
**Abdominal CT**	55 (29)	90,65	28	39	33	90,00	2,00	300,00

Abbreviations: Sed rate, sedimentation rate; AST, aspartate aminotransferase; ALT, Alanine transaminase; GGT, gamma-glutamyl transferase; FOBT, fecal occult blood test (3 samples); CEA, carcinoembryonic antigen; PSA, prostate specific antigen; CT, computed tomography. All values are in 2012 euros. Diagnostic and laboratory tests are shown in ascending order of cost.

* Within 25% of the true cost

Approximately one-quarter (24%; 95% confidence interval [CI]: 23%–25%) of all cost estimates were accurate to within 25% of the real cost (i.e., 25% for laboratory tests and 23% for other medical tests), with 55% (95% CI: 53–56%) overestimating the true cost and 21% (95% CI: 20%–22%) underestimating them.

Tests that were estimated to within 25% accuracy ranged from a high of 39% for GGT to a low of 11% for total cholesterol laboratory tests, and between 40% for echocardiogram and 9% of electrocardiogram for other medical tests (Table 3).

For most tests, the majority of family physicians overestimated costs. Only hemogram, platelets, urinalysis, proteinogram, and echocardiogram were underestimated. The most commonly overestimated were Pap smear, electrocardiogram, CEA, flexible sigmoidoscopy, and sedimentation rate.

We observed a tendency to overestimate all tests, and to do this more for medical tests (average 61%, 95% CI: 59%–63%) than for laboratory tests (average 49%, 95% CI: 48%–51%). We did not find a significant correlation between the true test cost and estimated costs accurate to within 25%.

**Fig 2:**
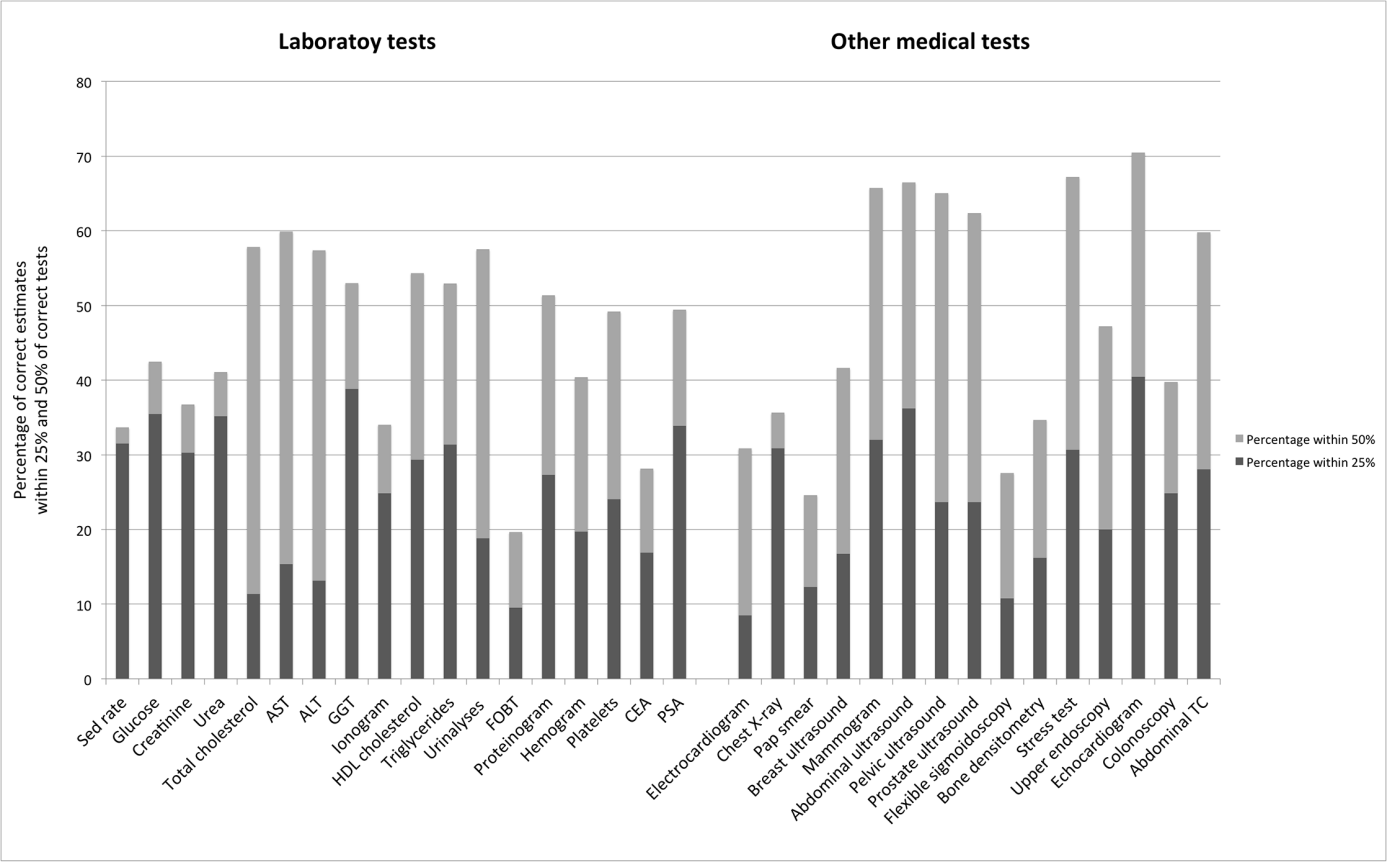
Accuracy of estimates for laboratory and other medical tests.

Fig 2 shows the percentage of estimates that were accurate to within 25% and 50% of true cost for laboratory and other medical tests. Overall, fewer than half (47%; 95% CI: 46%–48%) were correct to within 50%.

Awareness of cost did not correlate with gender or age, with the exception of colonoscopy cost, where family physicians who were at least 50 years old had a better estimate than younger physicians (30% compared to 15%, p = .025). Family physicians working in rural areas provided better estimates for sedimentation rates than urban physicians (42% compared to 26%, p = .023) and breast ultrasounds (26% compared to 12%, p = .012), and family physicians working in urban areas provided better estimates for type II urine costs than rural physicians (7% compared to 25%, p = .002).

Regarding family physicians’ beliefs regarding the cost of diagnostic and laboratory tests (Table 4), 53% thought they did not have or were uncertain about whether or not they had adequate knowledge about this issue, and only 7% stated that they felt they had received adequate education on diagnostic and laboratory test costs. However, the majority of the family physicians (82%) said that they felt that they had adequate access to information about diagnostic and laboratory test costs. With regard to the influence of test costs on ordering decisions, 33% indicated that cost did not influence their decisions, while 27% were uncertain.

**Table 4:** Family physician level of agreement about health care costs.

Statement	Agree	Undecided	Median
	(%, 95% CI)	(%, 95% CI)	
Diagnostic and laboratory test costs influence my decision when placing orders	33 (27–39)	27 (21–32)	3
I have adequate knowledge about diagnostic and laboratory test costs	47 (41–53)	29 (23–35)	3
I have received adequate education on the diagnostic and laboratory test costs	7 (4–10)	7 (4–10)	1
I have adequate access to information about diagnostic and laboratory test costs	82 (77–87)	5 (2–8)	4

Likert Scale: 1- totally agree, 2- agree, 3-undecided, 4-disagree, 5-strongly disagree

The level of agreement with the statements “I have adequate knowledge of the diagnostic and laboratory test costs,” and “I have adequate access to information on the diagnostic and laboratory test costs” was significantly associated with accuracy of cost estimates to within 25% (Table 5). In fact, those who agreed or strongly agreed with the statement “I have adequate knowledge of the laboratory and medical test costs” correctly estimated the median price of seven tests, whereas those who disagreed only correctly estimated the median price of five tests and those who strongly disagreed only correctly estimated the price of one test (p = .030). Those who agreed or strongly agreed with the statement “I have adequate access to information on the diagnostic and laboratory test costs” correctly estimated the median price of six tests, whereas those who disagreed only correctly estimated the price of five, and those who strongly agreed correctly estimated the price of zero tests (p = .011).

**Table 5:** Median number of tests in which doctors correctly estimated test costs to within 25%, by level of agreement with statements about health care costs.

Statement	Strongly agree	Agree	Undecided	Disagree	Strongly disagree	P value*
Diagnostic and laboratory test costs influence my decision when placing orders.	5	4	6	6	7	0.140
I have adequate knowledge of diagnostic and laboratory test costs.	7	7	4	5	1	0.030
I have received adequate education on diagnostic and laboratory test costs.	6	8	8	5	5	0.124
I have adequate access to information on diagnostic and laboratory test costs.	6	6	4	5	0	0.011

*As assessed by Kruskal-Wallis test

## Discussion

The results from our survey show that Portuguese family physicians feel that they do not have adequate knowledge about diagnostic and laboratory test costs, and, in fact, most physicians could not estimate these costs; fewer than one-quarter of all cost estimations were within 25% of the true value, and only 47% of cost estimates fell within 50% of the true value. There was no difference between laboratory tests and other medical tests. Of the 33 diagnostic and laboratory tests, only five had correct response rates greater than 35%: glucose, urea, GGT, abdominal ultrasound, and echocardiogram. These results are similar to previous studies, in which only a third of diagnostic and laboratory tests cost estimates fell between 20% and 25% of the true cost [10][11][12], and only 50% were within 50% of the true cost [12].

The majority of Portuguese family physicians overestimated test costs, particularly the non-laboratory tests, but the tendency for responders to incorrectly estimate test costs maintained regardless of the real test costs. We found no correlation between true costs and the correct estimates to within 25%. These results differ from those obtained in other countries, where a tendency toward underestimation of the more expensive tests and overestimation of the less expensive tests has been observed [10][11][12][13].

Only a minority of family physicians in Portugal felt they had adequate education about this issue, which is in agreement with the literature [10][11]. The family physicians who felt that they had adequate knowledge about the costs and those who felt they had adequate access to information on diagnostic and laboratory test costs actually provide better estimates of the test costs. However, nearly all surveyed physicians believed that they had adequate access to information about diagnostic and laboratory test costs. This result was expected, given the previously mentioned modification to the laboratory tests ordering system software to include the one button easy-click access to the exact cost of each prescribed test. Despite this easy access, it seems that this subject arouses little interest among Portuguese family physicians and this tool does not appear to be used.

In our study, most family physicians believe that costs do not influence their decision when placing orders, which runs counter to the findings of other studies [10][11]. In fact, several other determinants likely affect physicians’ tendencies in test ordering: patients, health care environment, and other physicians’ factors such as age, sex, degree of specialization, geographic, location and practice setting, individual beliefs, experience, knowledge, fear of malpractice litigation, physician regret, financial incentives, and provision of written feedback by peers or employers[12]. For example, primary care physicians in health maintenance organization settings ordered more tests for social and symbolic reasons and to resolve tensions and conflicts related to time constraints and access problems [28].Whether or not improving knowledge on cost leads to a change in ordering behavior is a question that should be answered in future research.

Recent strategies in Portugal to change ordering behavior include giving feedback to family physicians about their resource utilization through an indicator called “average cost of complementary diagnostic and therapeutic prescribed by user”; annual targets are set in order to lower costs for the NHS. The most recent data shows progressive decreases[7] [9], which in turn shows the influence that the costs may have on Portuguese family physicians. In addition, several studies have shown a significant decrease in test ordering after physicians were provided with information about test costs [13][14][15][16][17][18]. However, in prospective randomized controlled trials where physicians were randomized to either see test costs or cost information was withheld, the intervention had no substantial impact on test ordering[22].

These results show the need for better education. We believe that interventions that use multiple educational strategies will be more successful in achieving positive outcomes, as seen in recent studies[19] [29]. These might include educational programs, clinical guidelines[20][21], computer-based ordering[22][23], feedback and audits[15], physician incentives, and fundholding or formulary restrictions. However, we must also remember that some of these strategies might have a negative impact on health outcomes[30] [31], and this could lead to higher, long-term costs to the health system.

Although Portuguese family physicians in the NHS have access to one easy-click button that informs them of the exact cost of each prescribed test, it might be more effective to show the costs by default instead of depending upon physicians to click on the button. Strategies such as the automatic appearance of the cost of each test, pop-up reminders regarding costs, suggestions for cost savings, and the information of total cost for that patient could be implemented [32].

Awareness of costs was not associated with age, gender, or workplace, with the exception of a few tests. Previous studies found no significant differences in cost awareness among physicians, among different specialties, among physicians from diverse practice settings, faculty physicians, non-faculty physicians, residents, and students [10][32]. Country, year of study, and level of training did not impact accuracy[12] [33]. These findings have implications for program directors at medical schools, residencies, and post-graduate programs [10].

One of the strengths of our study is the representativeness of our sample, which allows us to generalize to all Portuguese family physicians. Another positive aspect of our study is the comparability of the cost estimation methodology with other international studies.

However, our research also has several limitations. A relatively high percentage of physicians did not answer the questions about the cost of diagnostic and laboratory tests, leading to possible response bias. This may be an indicator of physician frustration with their lack of knowledge about costs. However, the similarity of our results to those of previous investigators suggests the results are valid. In using a percentage range, the correct range of estimations for expensive tests is larger than that for inexpensive tests. This did not seem to have much impact, because there is homogeneity of errors from the more expensive to less expensive tests.

## Conclusion

Portuguese family physicians have limited awareness of the cost of diagnostic and laboratory tests, and our results demonstrate a need for better education in this area. Further research should focus on identifying whether interventions on cost knowledge actually change ordering behavior, on identifying optimal methods to disseminate cost information, and on improving the cost-effectiveness of care.

## Supporting Information

S1 Appendix (XLSX)

S2 Appendix (DOCX)
